# Danger signal uric acid is involved in ventilator-induced lung injury pathogenesis

**DOI:** 10.1186/cc9612

**Published:** 2011-03-11

**Authors:** M Kuipers, H Aslami, T Van der Poll, M Schultz, C Wieland

**Affiliations:** 1 Academic Medical Centre, Amsterdam, the Netherlands

## Introduction

Endogenous molecules released during tissue injury can trigger an innate immune response and are termed damage-associated molecular patterns (DAMPs). Uric acid is considered an important DAMP and causes acute lung inflammation when administered locally. The exact role of the innate immune response in ventilator-induced lung injury (VILI) is not yet completely understood. We hypothesized that uric acid is released during VILI and that reduction of uric acid levels attenuates lung injury induced by short-term mechanical ventilation (MV).

## Methods

Uric acid levels in bronchoalveolar lavage fluid (BALF) of wildtype C57BL/6 mice ventilated for 5 hours with low tidal volume (LVT ~7.5 ml/kg) or high tidal volume (HVT ~15 ml/kg) and spontaneously breathing mice were determined. In addition, mice were treated with allopurinol (25 mg/kg; inhibits uric acid synthesis) or uricase (0.2 mg/kg; degrades uric acid) or vehicle (10% DMSO), 1 hour before start of HVT MV. Endpoints of VILI were lung wet/dry ratio, total protein, IgM and sRAGE concentrations in BALF as well as neutrophil influx and pulmonary cytokine and chemokine levels.

## Results

Injurious MV leads to uric acid release in BALF of previously healthy mice. HVT ventilation significantly increased all endpoints of VILI as compared with the unventilated control group. Allopurinol and uricase treatment significantly decreased the wet/dry ratio and alveolar protein leak as compared with the HVT ventilated vehicle-treated group. IgM levels were also significantly lower in the allopurinol-treated group indicating protection of alveolar barrier function. Reduction of lung injury by allopurinol and uricase treatment was also demonstrated by the reduction of sRAGE concentrations, a marker of alveolar type I cell injury. Interestingly, treatment in the HVT group with allopurinol or uricase did not significantly reduce neutrophil influx or cytokine and chemokine levels.

## Conclusions

The danger signal uric acid is released due to injurious mechanical ventilation. Reduction of uric acid concentrations with allopurinol or uricase decreased VILI and specifically epithelial injury and alveolar barrier dysfunction

